# A weighting approach for judging the effect of patient strata on high-dimensional risk prediction signatures

**DOI:** 10.1186/s12859-015-0716-8

**Published:** 2015-09-15

**Authors:** Veronika Weyer, Harald Binder

**Affiliations:** Institute of Medical Biostatistics, Epidemiology and Informatics (IMBEI), University Medical Center Mainz, Johannes Gutenberg-University Mainz, Obere Zahlbacher Strasse 69, Mainz, Germany

**Keywords:** Weighted regression, Subgroup analysis, High-dimensional data, Time-to-event endpoint

## Abstract

**Background:**

High-dimensional molecular measurements, e.g. gene expression data, can be linked to clinical time-to-event endpoints by Cox regression models and regularized estimation approaches, such as componentwise boosting, and can incorporate a large number of covariates as well as provide variable selection. If there is heterogeneity due to known patient subgroups, a stratified Cox model allows for separate baseline hazards in each subgroup. Variable selection will still depend on the relative stratum sizes in the data, which might be a convenience sample and not representative for future applications. Such effects need to be systematically investigated and could even help to more reliably identify components of risk prediction signatures.

**Results:**

Correspondingly, we propose a weighted regression approach based on componentwise likelihood-based boosting which is implemented in the R package CoxBoost (https://github.com/binderh/CoxBoost). This approach focuses on building a risk prediction signature for a specific stratum by down-weighting the observations from the other strata using a range of weights. Stability of selection for specific covariates as a function of the weights is investigated by resampling inclusion frequencies, and two types of corresponding visualizations are suggested. This is illustrated for two applications with methylation and gene expression measurements from cancer patients.

**Conclusion:**

The proposed approach is meant to point out components of risk prediction signatures that are specific to the stratum of interest and components that are also important to other strata. Performance is mostly improved by incorporating down-weighted information from the other strata. This suggests more general usefulness for risk prediction signature development in data with heterogeneity due to known subgroups.

## Background

In high-dimensional data with time-to-event endpoints, regression models can provide risk prediction. For example, a Cox proportional hazards regression model could be used to investigate if there is an association between covariates, such as DNA methylation or gene expression data, and clinical endpoints, such as death in cancer patients. An automatic variable selection procedure, such as the Lasso ([1]) or componentwise likelihood-based boosting [2, 3] for regularized regression with variable selection, can be used to identify a small number of potentially important covariates, thus developing a risk prediction signature. With a Cox model, heterogeneity in the sample of patients due to known subgroups can be taken into account by allowing for different strata, each with its own baseline hazard. For example, in cancer studies, patients with different cytogenetic profiles may be assigned to different strata. However, when the effect of the different covariates varies between the strata, estimating regression coefficients will result in a weighted average. This is sensitive to the relative size of the strata in the data, which might be a convenience sample and not representative for future applications. In turn, this will also affect variable selection.

We propose an approach that actively controls the extent to which each stratum contributes to the variable selection and estimation of regression coefficients. Specifically, we introduce a weighted estimation approach for a stratified Cox model based on componentwise likelihood-based boosting that focuses on a specific stratum and down-weights the observations in the other strata. Observations in the stratum of interest each receive a weight of one, and observations in the other strata receive a fixed weight between zero and one in the partial log-likelihood.

One aim of this approach is to potentially increase variable selection stability compared to subgroup analysis for the stratum of interest. This will be particularly important when considering a large number of measurements (e.g. 20,000 gene expression values or more than 400,000 DNA methylation CpG measurements) for a limited number of patients. Especially for methylation data, where more than 400,000 CpG covariates are examined, signals in the data could be small. In particular for studies with less than 200 or 300 patients, the loss of power due to subgroup analysis for one stratum, for example one tumor stage, will be very problematic due to the limited amount of information. For relatively rare cancers, such as acute myeloid leukemia (AML), the size of even the largest available cohorts will be limited (e.g. to a few hundred cases for AML), making joint analysis of subgroups with known differences, e.g. different cytogenetic groups, a pragmatically attractive option. It is then important to gauge potential downsides and beneficial effects of a global joint analysis. In this situation, a weighted approach can provide fine-grained intermediate steps between the subgroup analysis and the global joint analysis. Specifically, we will investigate the effects on variable selection stability, as indicated by resampling inclusion frequencies [4], as a function of the weights and proposed tools for visualization. These aim to identify clusters of variables that either are important only in the stratum of interest or are also important to some extent in the other strata.

There are several statistical techniques that are based on re-weighting of observations or strata at a technical level. In epidemiological settings, re-weighting of strata is used to standardize results from one data set to a specific reference population (see for example [5]). This is typically done for crude rates or similar measures, but not for regression models. Weighted estimation for regression models is, for example, motivated by the approach by Simon [6] for obtaining treatment effect estimates in a clinical setting. Specifically, Simon [6] proposed a Bayesian approach for performing a subgroup analysis on the effect of a single covariate in a time-to-event setting that results in a closed-form solution for the estimates taking the form of weighted regression. Thus, our approach could be seen as an extension of Simon’s weighted regression approach, specifically introducing variable selection for a large number of measurements. Tutz and Binder [7], Binder, et al. [8] propose a weighted approach for variable selection, but without pre-defined strata and for settings with binary endpoints. While separate regression parameters might be estimated for each stratum in a sample, [9], the approaches of [6, 8, 10] borrow information from similar individuals for when the analysis focuses on a specific stratum. A similar reasoning is also used within the context of ROC curve estimation in the approach by [11], where a weighted estimate is used for the population-specific positive predictive value (PPV) and negative predictive value (NPV). The ROC curves are derived from a weighted average of the ROC curves of a “target population” and an “auxiliary population”. The estimate of the PPV and the NPV is then based on a weighted average of the ROC curves from both populations. Our proposal is built on the same general idea of borrowing information from other strata, but specifically focuses on variable selection stability for risk prediction signatures.

In the first Section, we introduce two application examples: One with kidney renal clear cell carcinoma (KIRC) and one with acute myeloid leukemia (AML) data. In the methods Section, the details of our weighted approach based on componentwise likelihood-based boosting for variable selection are given as well as two tools for visualizing resampling inclusion frequencies as a function of the weights: The *stability trajectories* and the *weight-frequency map*. The next Section presents the results for the two real data examples. We provide concluding remarks in the discussion Section.

## Gene expression and methylation applications

In a first application, we consider gene expression data from patients with acute myeloid leukemia (AML). There is a total of *n*=134 patients with two cytogenetic risk groups with different types of prognoses, normal/intermediate risk (*n*=98) and poor prognosis (*n*=36). The aim is to predict survival beyond the cytogenetic information, where 66 deaths were observed in the normal group and 26 deaths in the poor prognosis group. The focus is on developing a subgroup signature for patients from the normal/intermediate group based on *p*=18,714 (log-transformed) RNA-Seq gene expression measurements. In addition to the cytogenetic risk category, the information on different clinical covariates, age at diagnosis, gender, and mutation status of the FLT3 gene are available.

In a second application, we consider DNA methylation data from *n*=261 patients suffering from kidney renal clear cell carcinoma (KIRC) to predict survival. There are two strata, one with 206 stage I - III patients and a second with 55 stage IV patients; 48 deaths were observed in the former and 42 deaths in the latter. For prediction, Illumina 450 K methylation array data are available, comprising *p*=485,577 CpG covariates. In the analysis, M-values were used, which are calculated as the log2 ratio of the intensities of methylated probe versus unmethylated probe intensities (see [12]). For each of the 261 patients, there are two important clinical covariates available: age and the cancer side (right kidney (*n*=140) or left kidney (*n*=121)). To develop a CpG signature, we focus on the stage I - III patients. The tumors at stage IV tend to have already metastasized, unlike their lower stage counterparts.

Both data sets are freely availabe at “The Genome Cancer Atlas (TCGA)” (https://tcga-data.nci.nih.gov/tcga/).

## Methods

In the following, we consider a time-to-event setting with observations (*t*_*i*_,*δ*_*i*_,*x*_*i*_,*s*_*i*_), *i*=1,…,*n*, where *t*_*i*_ is the observed time for individual *i*, which is given by *t*_*i*_= min(*T*_*i*_,*C*_*i*_), where *T*_*i*_ is the event time and *C*_*i*_ is the censoring time. The status indicator *δ*_*i*_ takes the value 1 if an event occurred at that time and 0 if the observation has been censored. *x*_*i*_=(*x*_*i*1_,…,*x*_*ip*_)^*T*^ is the vector of covariates observed at time zero (baseline), and *s*_*i*_∈{1,…,*S*} indicates the stratum the individual belongs to.

Similar to many approaches for high-dimensional survival data, in the following we will consider a Cox proportional hazards model, specifically a stratified version

(1) $$ \lambda^{(s)}(t|x_{i}) = \lambda_{0}^{(s)}(t)\exp(\eta_{i}) = \lambda_{0}^{(s)}(t)\exp\left({x_{i}^{T}}\beta\right),  $$

where $\lambda _{0}^{(s)}(t), s=1,\ldots,S$ are the baseline hazard functions for the *S* different strata of individuals. The parameter vector *β*=(*β*_1_,..,*β*_*p*_)^*T*^ can be estimated without having to estimate the baseline hazards by maximizing a partial likelihood.

The aim is to estimate the parameter vector *β* in a setting with *p*>>*n* with a focus on individuals from one specific stratum *s*_*i*_=1, while still retaining some information from the other strata. In the following, we introduce a weighted partial likelihood before considering estimation and variable selection in a high-dimensional setting based on this.

The data used in this study was from the TCGA data portal, this data is freely accessible therefore did not require ethical approval.

### Weighted partial likelihood

The estimate of the parameter vector *β* of a Cox proportional hazards model, where strata are taken into account, can be obtained by maximizing the stratified partial log-likelihood function. We modify the latter by introducing weights *w*_*i*_∈[0;1],*i*=1,…,*n* as follows:

(2) $$\begin{array}{@{}rcl@{}} {}l(\beta)&=&\sum_{s=1}^{S}\left[\sum_{i=1}^{n} I(s_{i} = s) w_{i}\delta_{i}\left({x_{i}^{T}}\beta\right.\right. \end{array} $$

(3) $$\begin{array}{@{}rcl@{}} {}&-&\left.\left.\log\left\{\sum_{k=1}^{n} I(s_{k} = s) w_{k}I(t_{i}\leq t_{k})\exp\left({x_{k}^{T}}\beta\right)\right\}\!\right)\!\right], \end{array} $$

where *I*(·) is an indicator function taking a value of 1 if its argument is true and 0 otherwise.

While this general form of the weighted likelihood would allow for different weights for each individual, we use a simpler scheme

$$w_{i} = \left\{ \begin{array}{ll} 1 & \text{if \(s_{i} = 1\)}\\ w & \text{otherwise} \end{array} \right., $$

where *w*∈[0;1] is the main tuning parameter of our weighted regression approach. The weighted partial log-likelihood takes the form of a weighted sum of standard per-stratum Cox regression partial log-likelihoods. For *w*=1, the standard stratified partial log-likelihood is recovered, corresponding to a global analysis. In all other cases, i.e. *w*∈[0;1[, the observations from the other strata are retained but down-weighted, where a subgroup analysis is obtained for *w*=0. Note that this will only be beneficial if several of the true non-zero effects in the strata have the same sign.

### Weighted componentwise likelihood based boosting

For parameter estimation and variable selection in a high-dimensional setting, i.e. *p*>>*n*, a componentwise likelihood-based boosting approach, introduced by [2], is adapted for weighted regression. This approach regularizes estimates such that many elements of the estimated parameter vector will be equal to zero, thus performing variable selection. Estimation is performed in a potentially large number of steps as follows:

1. Start with estimate $\hat {\beta }^{(0)}=(0,\ldots, 0)^{T}$ and offset $\hat \eta ^{(0)}_{i} = 0$.
2. For each boosting step *m*=1,…,*M*:
  - Consider univariate candidate models for each covariate *j*=1,…,*p* using the linear predictor
    $$\eta_{i} = \hat\eta^{(m-1)}_{i} + \gamma^{(m)}_{j} x_{ij} $$
    and a weighted penalized partial log-likelihood
    $$l\left(\gamma_{j}^{(m)}\right)-\rho\gamma^{(m)}_{j}, $$
    where *ρ*≥0 is a penalty parameter that needs to be set.
  - Determine the best update candidate *j* ^∗^ according to the penalized score statistic
    (4) $$ \frac{(U(0))^{2}}{I(0)}  $$
    where
    $$\begin{array}{@{}rcl@{}} U(\gamma) &=& \frac{\partial l}{\partial \gamma }(\gamma)\\ &=& \sum_{s=1}^{S} \left\{\sum_{i=1}^{n} I(s_{i} = s) w_{i}\delta_{i}\ast \left[{\vphantom{\left.\frac{\sum_{k=1}^{n} I(s_{k} = s) w_{k}I(t_{i}\leq t_{k}) \hat\eta^{(m-1)}_{i} x_{k} \exp\left(\hat\eta^{(m-1)}_{k} +\gamma^{(m)} x_{k}\right)}{\sum_{k=1}^{n} I(s_{k} = s) w_{k}I(t_{i}\leq t_{k})\exp\left(\hat\eta^{(m-1)}_{k} +\gamma^{(m)} x_{k}\right)}\right]}}x_{i}^{T}\right.\right.\\ &-& \left. \left.\frac{\sum_{k=1}^{n} I(s_{k} = s) w_{k}I(t_{i}\leq t_{k}) \hat\eta^{(m-1)}_{i} x_{k} \exp\left(\hat\eta^{(m-1)}_{k} +\gamma^{(m)} x_{k}\right)}{\sum_{k=1}^{n} I(s_{k} = s) w_{k}I(t_{i}\leq t_{k})\exp\left(\hat\eta^{(m-1)}_{k} +\gamma^{(m)} x_{k}\right)}\right]\right\} \end{array} $$
    is the weighted and stratified score function and
    $$\begin{array}{@{}rcl@{}} I(\gamma)&= \frac{\partial^{2} l}{\partial \gamma \partial \gamma^{t}}(\gamma)\\ & = \sum_{s=1}^{S} \left\{{\vphantom{\left. \left(\frac{\sum_{k=1}^{n} I(s_{k} = s) w_{k}I(t_{i}\leq t_{k}) \hat\eta^{(m-1)}_{i} x_{k} \exp\left(\hat\eta^{(m-1)}_{k} +\gamma^{(m)} x_{k}\right)}{\sum_{k=1}^{n} I(s_{k} = s) w_{k}I(t_{i}\leq t_{k})\exp\left(\hat\eta^{(m-1)}_{k} +\gamma^{(m)} x_{k}\right)}\right)^{2}\right]}}-\sum_{i=1}^{n}I(s_{i} = s)w_{i}\delta_{i} \right.\\ &*\left. \left[\frac{\sum_{k=1}^{n} I(s_{k} = s) w_{k}I(t_{i}\leq t_{k}) \hat\eta^{(m-1)}_{i} {x_{k}^{2}} \exp\left(\hat\eta^{(m-1)}_{k} +\gamma^{(m)} x_{k}\right)}{\sum_{k=1}^{n} I(s_{k} = s) w_{k}I(t_{i}\leq t_{k})\exp\left(\hat\eta^{(m-1)}_{k} +\gamma^{(m)} x_{k}\right)} \right. \right. \\ &-\left. \left. \left(\frac{\sum_{k=1}^{n} I(s_{k} = s) w_{k}I(t_{i}\leq t_{k}) \hat\eta^{(m-1)}_{i} x_{k} \exp\left(\hat\eta^{(m-1)}_{k} +\gamma^{(m)} x_{k}\right)}{\sum_{k=1}^{n} I(s_{k} = s) w_{k}I(t_{i}\leq t_{k})\exp\left(\hat\eta^{(m-1)}_{k} +\gamma^{(m)} x_{k}\right)}\right)^{2}\right]\right\} \end{array} $$
    is the weighted and stratified penalized Fisher information. An estimate from one Newton-Raphson step is obtained as
    $$\begin{array}{@{}rcl@{}} \widehat{\gamma}_{j^{*}}^{(m)}=\frac{U(\widehat{\gamma})}{I(\widehat{\gamma})+\rho}. \end{array} $$
  - Update
    $$ \widehat{\beta_{j}}^{(m)}=\left\{ \begin{array}{ll} \widehat{\beta_{j}}^{(m-1)}+\widehat{\gamma}_{j}^{(m)} & \text{if \(j=j^{*}\)}\\ \widehat{\beta_{j}}^{(m-1)} & \text{if \(j\neq j^{*}\)} \end{array} \right., $$
    and
    $$\hat\eta^{(m)}_{i} ={x_{i}^{T}}\hat\beta^{(m)} \,\,\,\, i = 1, \ldots, n.$$

The componentwise boosting approach gives a special role to covariates that should always be included, e.g. clinical covariates for adjustment, when performing variable selection for high-dimensional molecular measurements. Before each boosting step, the estimates for such mandatory covariates are updated with standard maximum partial likelihood methods. For the proposed stratified approach, we also allow for different regression coefficients for each stratum for all mandatory, unpenalized variables. The number of boosting steps *M* must be carefully chosen because it is a critical parameter that controls the complexity of the model. In the following, the number of boosting steps is empirically chosen by 10-fold cross validation. The penalty parameter *ρ* is less important as long as it is chosen so that it is large enough. In the following, we use $\sum _{i} \delta _{i} (1/0.02 - 1)$, which will result in updates approximately the size of 0.02 times the maximum partial likelihood estimates.

### Visualizing stability for different weights

While the weight in the approach above could be considered as a tuning parameter, requiring selection of one fixed value by an approach such as cross-validation, we propose to investigate the effects of different weights to potentially identify covariates that are primarily important for the stratum of interest only as well as groups of covariates that also have some effect in the other strata; a joint analysis might therefore be beneficial.

Specifically, the benefit of weighted inclusion of observations from other strata is quantified by variable selection stability. We evaluate selection stability by using resampling techniques, and determine per-covariate resampling inclusion frequencies (RIFs) [13, 14], i.e. the proportion of resampling data sets where a covariate receives a non-zero parameter estimate by the weighted boosting approach. Therefore, we repeatedly randomly split the data into training data sets and test data sets, drawing 0.632 *n* observations without replacement for each resampling data set, as is considered for high-dimensional molecular data in [4].

**Fig. 1:**
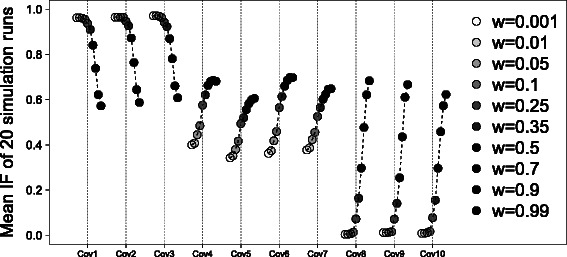
Stability trajectories for the simulation study. Here, the inclusion frequencies for the simulation scenario for different weights are shown
(ii) *Weight-frequency map*: This is a graphical tool for a larger number of covariates in the form of a heat map. Specifically, the RIFs are presented here for different weights and different covariates in a heat map, where covariates are clustered based on the Pearson correlation of RIFs across weights. Lighter shades of gray indicate a more stable variable selection. The aim of this plot is to detect kinds of clusters of covariates. This aims to reveal different groups of covariates that are associated with either only one or with more strata.
  In Fig. 2 the weight-frequency map for the artificial data is presented for covariates with an inclusion frequency above 0.03 for some of the weights. The weight-frequency map indicates different clusters that are in accordance with the underlying true structure. One cluster of covariates contains Cov4 to Cov7; these covariates are simulated as having an effect of 0.5 in both groups and show, as expected, the best results for high weights. Another cluster contains Cov8 to Cov10, which have an effect in the second subgroup and the non-analyzed subgroup. These covariates only receive large inclusion frequencies when the complete information of the second subgroup is used. A third cluster includes Cov1 to Cov3, which are simulated as having an effect in the first subgroup and the analyzed subgroup. The IFs are relatively large for all weights, but are larger for the subgroup analysis, as expected. All other covariates have no effect and correspondingly are not stably selected with any of the weights using the componentwise boosting approach.

For the visualization of selection stability for different weights when using our weighted approach, we introduce two graphical tools:

(i) *Stability trajectories*: The stability trajectory plot is a graphical tool for a small set of stable covariates, i.e frequently selected covariates, that tracks stability as a function of weights. Specifically, we suggest plotting the results for covariates which have RIFs above 0.1 to 0.2 for some of the weights. The variable selection stability is presented in the form of RIFs on the y-axis. The different weights are denoted with different shades of gray and the visually “best” weight on average for the different covariates shown is marked with a triangle (only for real data sets). All RIFs for different weights from one covariate are connected with each other in this graphic with a dotted line.
  For illustration, we considered artificial time-to-event data with a total of *n* = 250 individuals and *p* = 1000 uncorrelated covariates. We used exponentially distributed survival and censoring times (see [15]), each with a scale parameter of $\frac {1}{20}$, and simulated two groups of equal size from a binomial distribution (with a probability of 0.5). Instead of resampling, data were generated 20 times. We assumed that 10 covariates have an effect in the first, the second or in both subgroups, and that all other covariates have no effect. For the first three covariates, we assumed an effect of one in subgroup 1 (*β*_*i*1_=1 for i=1,2,3) and zero in subgroup 2 (*β*_*i*2_=0 for i=1,2,3), for the next four covariates an effect of 0.5 in both subgroups (*β*_*i*1_=*β*_*i*2_=0.5 for i=4,5,6,7) and for covariates eight to ten an effect of zero in subgroup 1 (*β*_*i*1_=0 for i=8,9,10) and one in subgroup 2 (*β*_*i*2_=1 for i=8,9,10). In this simulation scenario, the mean effect over both subgroups is 0.5 for the first 10 covariates of 1000. All other covariates are simulated as having no effect on the survival time.
  In Fig. 1 the stability trajectories are presented. On the x-axis, the first 10 covariates which have an effect in subgroup 1 (Cov1 to Cov3), in subgroup 2 (Cov4 to Cov7) or in both subgroups (Cov8 to Cov10) are shown. On the y-axis, the mean IFs over 20 simulation runs are plotted. The different shades of gray indicate the different weights, from a weight of 0.001 to 0.99. Lighter gray indicates that only a small amount information of the second subgroup is used in the analysis. The darker the color, the more information from the second subgroup is used in the analysis. Different weights result in widely different IFs, i.e. there is a distinct effect of weighting. Overall, the three covariates one to three which have an effect of one in the first and analyzed subgroup receive the largest inclusion frequencies with weights around 0.001 to 0.1. For covariates four to ten, the global analysis, as expected, results in the best IFs. Almost excluded from the subgroup analysis are the covariates that only have an effect in subgroup 2, which is the subgroup that was not analyzed. As a consequence of simulating an overall mean effect of 0.5 for the global analysis, covariates one to ten have similar inclusion frequencies here. It is not easy to select the best average weight, but a weight equal to zero would mean that covariates four to seven are not stably selected, although there is an effect in subgroup 1 and they should be selected from the model. Therefore a weight of zero should not be chosen.

**Fig. 2:**
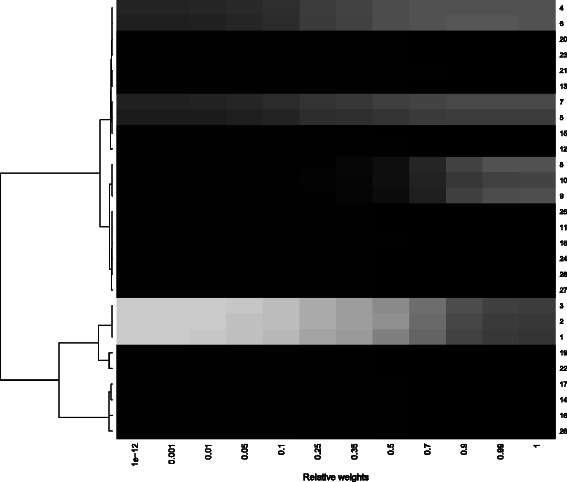
Weight-frequency map for the simulation study. This figure shows a heat map for the simulation scenario for different weights based on the correlation distance

### Implementation of the visualization tools

To provide the proposed visualization tools in a readily accessible way, we extended the R package CoxBoost. To our knowledge the latter is the only implementation allowing for stratified, weighted regularized regression for time-to-event data, i.e. this is the natural candidate for extension. Specifically, we added a new function resample.CoxBoost, which builds on the CoxBoost function for calculating resampling inclusion frequencies for different weights. The two proposed types of visualizations for presenting the resampling inclusion frequencies for different weights can be obtained applying the functions stabtrajec or weightfreqmap to the result of resample.CoxBoost. The implementation, containing the functions resample.CoxBoost, stabtrajec, and weightfreqmap, will be made available on CRAN, but is already provided in the development version of the package CoxBoost on GitHub (https://github.com/binderh/CoxBoost). The documentation there also provides examples for obtaining the proposed visualizations.

For illustrating compute time, we used simulated data with *n* = 400 individuals and 1000 covariates. Using 10 resampling data sets, computation took 6.5 h on a Intel Xeon E5-2680 2.8 GHz processor. The 10-fold cross-validation performed in each of the resampling data sets and for each of 10 weights as a default can be trivially parallelized, and this is also implemented in the package. Using 10 cores, computation finishes after 45 min.

## Results and discussion

### Application example results

#### Results for the AML data

For the AML data, we focused on the cytogenetic low risk category and developed a corresponding risk prediction signature. We included the whole gene expression data of *p* = 18,714 genes as candidate covariates in the analysis and used the cytogenetic risk classification (high risk and low risk) as a weighting and stratification factor. The clinical variables age, gender and FLT3 status were incorporated as mandatory variables. Because it is well known that RNA-Seq gene expression data are skewed, the log transformed data were used for the analysis.

All results are based on 100 resampling data sets. Here, both graphical tools, the stability track plot and the weight-frequency map, are presented to display the results of weighted regression with respect to the selection stability.

**Fig. 3:**
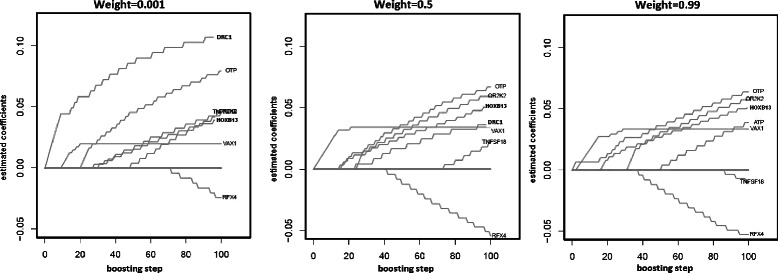
Coefficient paths for the AML data. Here, different coefficient paths for the AML data for three weights 0.001 (subgroup analysis), 0.5 (weighted model) and 0.99 (global analysis) are shown

In Fig. 3 the effect of variable selection for three weights is presented for the coefficient paths in the original data, i.e. the establishment of estimated parameters in the course of the boosting steps. The figure shows coefficient paths for three different weights, one with a very small weight of 0.001, one with a weight near one and one with a weight of 0.5; this represents here a weighted model where half of the information of the second subgroup is used in the analysis. The number of boosting steps are normally chosen by 10-fold cross validation. However, for purposes of illustration, 100 steps are presented on the x-axis, and the parameter estimates are plotted on the y-axis. At the end of each boosting step, the covariate that improves the model the most is updated. For the AML data, for example, the genes DRC1 for weight = 0.001, VAX1 for weight = 0.5 and OTP for weight 0.99 are updated in the first boosting step because they have the best score statistic. Many covariates which are not shown in this picture have regression coefficients of zero in all boosting steps and are not included in the final model. As boosting shrinks the regression coefficients of variables that do not improve the model to zero, only a small number of variables have a non-zero effect.

In the three different coefficient paths in Fig. 3, the gene DRC1 is selected from the subgroup model but not from the global analysis. Potentially this gene might only be associated with survival for the cytogenetically low risk patients. In addition, the gene HOXB13 is selected by all three models. The HOXB13 gene is a known predictor gene for different cancer types, such as breast cancer. We see that HOXB13 is selected from the weighted model (weight = 0.5) earlier in the procedure (around steps 15–18) than for the subgroup analysis (around step 25) or for the global model (around step 30), indicating that with weighted regression it might be easier to detect this gene. RFX4 is also selected in the subgroup analysis (weight = 0.001), although very late (around step 70). Additionally, RFX4 is also selected in the weighted model (weight = 0.5) and the global analysis (weight = 0.99). This suggests that with the additional information of the high risk patients, RFX4 can be better identified.

**Fig. 4:**
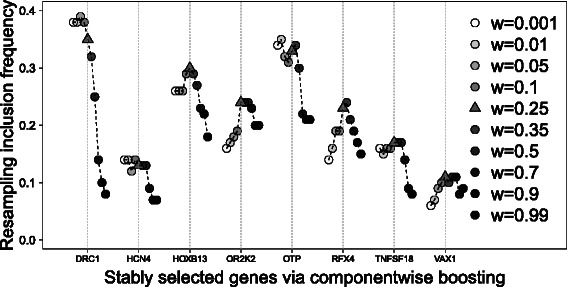
Stability trajectories for the AML data. This figure shows the resampling inclusion frequencies for stably selected genes with a RIF at least of 0.1 at any weight

Figure 4 shows the stability trajectories for the AML data, where the RIFs for eight genes which are selected with an RIF larger than 0.1 for some of the weights are presented. Overall we see that different weights result in widely different RIFs, similar to the artificial data above. There are genes with the highest RIF at low weights, but there are also genes where the highest RIF is at a weight different from zero and one. The weight that leads to the overall best variable selection stability is 0.25 and is marked with a triangle.

In addition to model stability, prediction performance was examined. Herewith, we want to ensure that we do not loose any prediction performance when using weighted regression. We consider prediction error curves, i.e. the Brier score ([16]), adapted for time-to-event endpoints ([17]). Specifically, we used the 0.632 + prediction error curve estimates ([18]) based on the 100 resampling data sets. We computed prediction error curves for the null model (Kaplan-Meier), for a model with only clinical covariates (age, gender and FLT3 status), and for the different weighted models with the gene features for the AML data with the clinical variables as mandatory variables. Furthermore, we calculated the integrated prediction error, which is the area under the different prediction error curves.

**Fig. 5:**
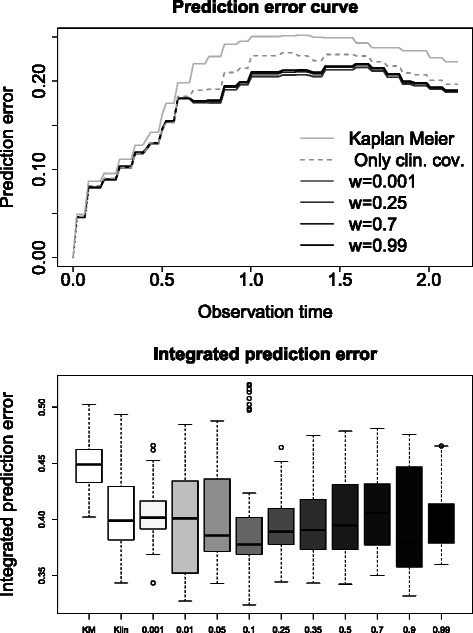
Prediction error curves for the AML data. Different prediction error curves based on the.632+ estimate (first figure) for the null model, for a model only with clinical covariates and for four different weighted models and integrated prediction error (second figure) are shown

In the top panel of Fig. 5, the prediction error curves based on the 0.632 + estimator for the low risk subgroup are shown. The weighted models and the model only with clinical covariates are seen to outperform the Kaplan-Meier estimate. The prediction error curves for large weights (0.7 and 0.99) are above those for the weighted models with lower weights, indicating somewhat better performance for the latter. The prediction error curve for the subgroup analysis and for a weight of 0.25 are located very close together and have the best prediction performance. So we can conclude that we do not loose any prediction performance with weighted regression for smaller weights. In the second figure of Fig. 5, boxplots for the integrated prediction error are shown for the different weighted models and the null model. The lowest integrated prediction error is at a weight of 0.1. This indicates that weighted regression for this AML data set is not only equivalent to the prediction error of the subgroup analysis, but might also perform better, at least by a small amount.

#### Results for the KIRC data

**Fig. 6:**
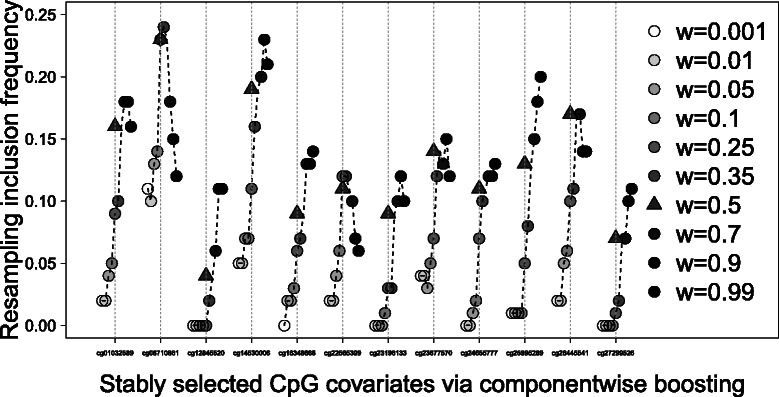
Stability trajectories for the KIRC data. Resampling inclusion frequencies for stably selected CpG covariates with a RIF at least of 0.1 at any weight

For the KIRC data, there were 485,577 CpG covariates overall that could be analyzed for an association with survival in tumor stages I to III. 89,512 CpG sites had to be excluded from the analysis because of missing values in all patients. The analyses were performed for only 396,065 CpG covariates. The model is adjusted for age and laterality (cancer side), which are included as mandatory variables. A heuristic approach was used for componentwise boosting to decrease computational demand [3]. Figure 6 shows the stability trajectories for the 12 CpG covariates that had a RIF between 0.1 and 1 for some of the weights. Qualitatively, the results are similar to the results of the AML data. Different weights result in widely different RIFs. Subgroup analysis is not well suited for developing a subgroup signature because the RIFs for low weights are very small. This indicates that weighted models with weights unequal to zero can improve the variable selection stability. Some CpG covariates received the best RIF for the global analysis (e.g., CpG cg12845520, cg16348668, cg24655777, and in particular cg25995289 and cg27299526). These CpG covariates might be associated with the survival time either in both subgroups or only in the subgroup that is not at the focus. Most of the CpG covariates have the highest RIF at a weight between the subgroup and the global analysis, which indicates the potential benefit of weighted regression. Visually, the best weight on average is 0.5, marked in Fig. 6 with a triangle. All CpG covariates shown in this stability trajectories have a large RIF at this weight or the largest RIF (cg26445541). This weight seems to be a good weight with respect to the best mean inclusion frequency over these stably selected covariates. With this weight, the important CpG covariates for the first stratum can be detected much better than the subgroup analysis would do.

**Fig. 7:**
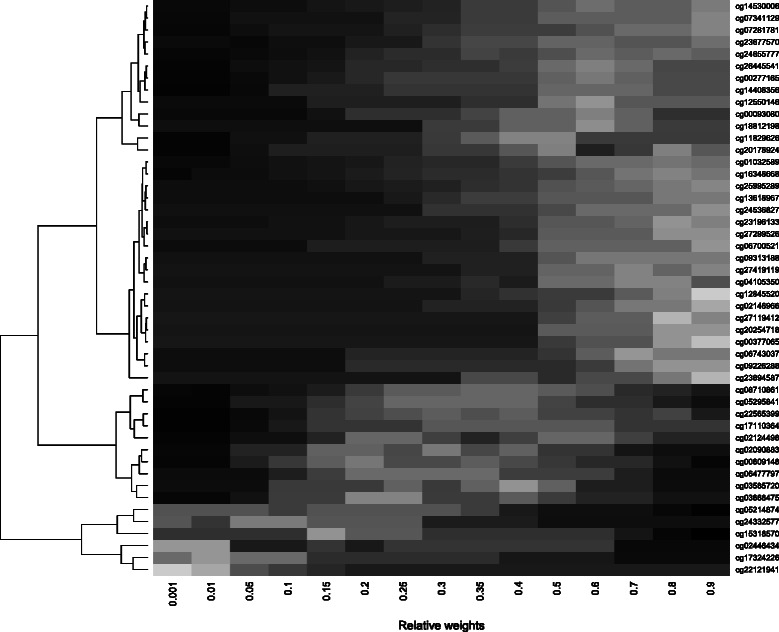
Weight-frequency map for the KIRC data. This figure shows a heat map for 15 different weights via boosting for stably selected CpG covariates between 0.05 and 1. Light gray color indicates good variable selection stability

To gain better insight in the RIF distribution, Fig. 7 shows the 48 covariates with a RIF of at least 0.05 for 15 different weights in the weight-frequency map. For some CpG covariates, there are lighter shades of gray for intermediate weights, indicating the best model stability for weighted regression. Different clusters are clearly discernible from this plot. In the top right there are the lighter shades of gray, whilst the bottom right contains the darker gray shades. In top right there are the covariates which are associated with both subgroups or possibly with the non-analyzed subgroup. In the bottom right there are the covariates which are only associated with the first and analyzed subgroup, because RIFs are not large for high weights, but rather are high for low weights. Overall, most of the covariates cannot be detected reliably with a subgroup analysis. This can be seen from the dark gray colors in the left of the heat map at low weights (except the last five CpG covariates). In the stability trajectories, a weight of 0.5 seems to be the largest on average. The weight-frequency map confirms this by showing the overall best RIFs (brightest gray at a weight of 0.5).

### Discussion and outlook

When analyzing gene expression or high throughput methylation data, for example from the 450 k Illumina methylation assay, a very large number of covariates are to be examined. To test the association of high-dimensional data with the survival time of patients, a variable selection can be performed to obtain a risk prediction signature for the patients. Often the entire study sample is not at the focus, but rather only patients in a specific subgroup. For example, different tumor stages or different cytogenetic risk profiles can divide the total study population into two or more different and, above all, heterogeneous sub-populations.

To account for this heterogeneity due to subgroups, we proposed a weighted approach for estimating a stratified Cox regression model based on componentwise boosting for automatic variable selection. With this weighted analysis, we want to account for heterogeneity in the study sample to increase the variable selection stability, as quantified by resampling inclusion frequencies, without a loss of prediction performance. To investigate the effects of different covariates in different strata, we proposed two visualization tools based on resampling inclusion frequencies: the stability trajectory plot and the weight-frequency map. The results of two application examples indicate that different weights do indeed affect the variable selection stability, as seen from resampling inclusion frequencies. Some genes can be identified better with the weighted model than with the subgroup analysis. There even may be groups of covariates that share the same weight-stability pattern, as indicated by the clustering in the weight-frequency map, which could potentially be useful to better understanding molecular processes relevant to certain sub-populations.

When choosing a weight of 0.25 or 0.5, we cannot say that this weighted model results in the largest RIFs for each CpG or gene covariate, but on average one can say that these weights seem to be the best in the real data examples. The prediction error for the AML data is neither better nor worse for a weighted model with a weight factor of 0.25, which is also a stable weight for model stability in comparison to the subgroup analysis. So with weighted regression, the prediction performance is not decreased, which makes weighted regression a very attractive approach.

We did not consider all available clinical parameters for defining potential strata, but focused on covariates related to the design of a study. For example, when AML data are analyzed, the cytogenetic risk category is a variable that would be used as a criterion for selecting patients for a study. With weighted regression, what happens when the composition of the study sample is somewhat different could potentially be anticipated.

## Conclusions

Overall our results show that different weights result in different model stabilities. The subgroup analysis, which is often used when analyzing subgroup effects, is not the best option, but middle weights of around 0.25 and 0.5 result in the best findings in the examples. We conclude that there is a positive effect gained by weighted regression.

Stratified weighted regression seems to be a useful technique for analyzing high-dimensional data with heterogeneity due to subgroups when focusing on developing a risk prediction signature for one specific stratum. While the current proposal was built on componentwise likelihood-based boosting, we expect that such an approach could also be adapted for other regularized regression approaches, such as the Lasso, but this requires implementations that allow for stratified weighted estimation for time-to-event endpoints, which are not available so far, to our knowledge. For componentwise boosting such an implementation is already provided by our package CoxBoost.
